# Peer review of "Asymmetric, biraphid diatoms from the Laurentian Great Lakes"

_PeerJ, doi:10.7717/peerj.14887_

## Round 0.1 · original submission · Major Revisions

Dear Dr. Reavie,

The reviewers checked your manuscript. They detected some weaknesses that must be addressed. In particular, they have concerns about images used to document the taxa and suggest the use of scanning electric microscopy images. Moreover, more generally reviewers ask for a deeper treatment of the taxa. So, I encourage you to improve the manuscript according to the tips of the reviewer.

Please, respond point-to-point to the comments of reviewers to speed up the process of revision. Once again, thank you for submitting your manuscript to PeerJ and we look forward to receiving your revision.

Sincerely,
Gabriele Casazza

·

Basic reporting

I did read this through a couple of times. It’s a valuable addition to knowledge of the diatoms found in the Laurentian Great Lakes. It was a little disappointing that much of the justification for the IDs is anecdotal rather than character oriented. For example, ‘Delicatophycus sp. Bell River 2’ has “This specimen, also from Bell River, has many of the characters of Delicatophycus neocaledonicus but has a too-fine striae count”. Yes, but what are those ‘many’ and why does this discount it being Delicatophycus neocaledonicus? And many of the specimens found are ‘matched’ to other published images, and so on and so forth.

The title might be: ‘Asymmetric, biraphid diatoms from the Laurentian Great
Lakes with a description of Cymbopleura subfrequens Reavie nov. sp.’

Although PeerJ have their own requirements, it would be useful to register this name at https://www.phycobank.org/

PeerJ state:

New Diatom Taxon: For diatom names there is no formal registration necessary but DiatomBase is part of WorMS, and can assign LSID(s) after the name has been published. We recommend that authors make sure their names are included in DiatomBase in a timely manner by forwarding their PDF, DOI or link to their PeerJ publication as soon as possible.

But Phycobank should be added too.

Some specifics are:

Line 24-5, “Despite being the largest surface freshwater system in the world, The Laurentian Great Lakes diatom flora is poorly defined”, poorly known, perhaps?

Line 45, “Laurentian Great Lakes diatoms have covered centrics and araphid taxa (Reavie & Kireta, 2015)”

Both ‘centrics and araphid’ taxa are known pretty much known to be non-monophyletic, I wouldn’t insist on this but it would be better to indicate that somehow, maybe like: “Laurentian Great Lakes diatoms have covered ‘centric’ and ‘araphid’ taxa (Reavie & Kireta, 2015)”

Line 101-2, “Preserved material and prepared microscope slides are currently stored in the Natural Resources Research Institute’s diatom collection at the University of Minnesota Duluth.”. The word ‘currently’ worries me a bit. Does that imply it might not be permanent? Is so, could some more permanent place be sought?

Lines 118-9 (and line 139), “In most cases I do not establish new taxa because of uncertainty about whether a given difference reflects variability in an existing species, and so additional specimen observations are recommended”. Just a comment: I’ve never quite understood this: so when does that action, “establishing new taxa”, take place? It implies that a certain quantity of information is required. Not sure how that could ever be established.

Lines 121et seq. “the lack of viable assignment to known species is a result of past reliance on (mostly) Eurasian concepts, such as the many published accounts from European authors. Now, we are fortunate to have an accelerating publication of North American diatoms, such as through the open-access Diatoms of North America portal (Spaulding et al., 2021; cited multiple times herein)”. Absolutely! And great work it is too – worth emphasising.

Line 206 (and elsewhere): A note on authors. It might be a pain, but data of this sort, taxon descriptions, are harvested by many online efforts. Here the author names can be a source of further confusion for those using them second hand. For example, Amphora calumetica is given as Amphora calumetica (Thomas) Peragallo. Thomas is a common name and there were two Peragallo’s. If the IPNI (https://www.ipni.org/) is followed, which it should be, it would be Amphora calumetica (B.W.Thomas) M. Peragallo. These changes will help when the data are used elsewhere. It will be a drag going through all the names, but worth it.

Line 237, “While having similarities to Amphora ovalis var. affinis, my specimens tended to be shorter than the minimum length described by Patrick & Reimer (1975)”. A picky comment but they’re not your specimens. “While having similarities to Amphora ovalis var. affinis, these specimens tended to be shorter…”

Line 258, “Because of a lack of detailed micrographs and large size of mu specimens, I am tentative about confirming this nomenclature for the Great Lakes”. I suppose ‘mu’ is a typo for ‘my’. Again, I’d suggest depersonalising the text. And it is not nomenclature (that’s the name), it is taxonomy that is being dealt with. Perhaps:

“Because there is a lack of detailed micrographs for Amphora macedoniensis and the Great Lake specimens are larger, assigning them to that species would be premature”. Or something like that.

Line 307, “Amphora pediculus/inariensis (Figs. 4MMMMM-VVVVV) This set of larger specimens illustrates that the distinction between the species is ambiguous. These specimens have length = 13-24 µm, width = 2.5-4 µm, dorsal striae = 18-24/10 µm”.
OK, but is this purely on size alone? You perhaps need to say more than this.

Line 311, Amphora siberica Skvortzow & Meyer.
The name was originally spelt Amphora sibirica. It might be best to retain that.

Line 358, Halamphora oligotraphenta (Lange-Bertalot) Levkov
You might need to clarify the text. Amphora oligotraphenta was a new name for Amphora veneta var. capitata Haworth as it could not be Amphora capitata as that name had been used by Brander (1933). I assume all of these -- Germain (1981), Patrick & Reimer (1975) and Krammer & Lange-Bertalot (1986) – refer to A. veneta.

Line 493, Cymbella sp. (Old Woman Creek, Lake Erie) (Fig. 6O)
“This challenging specimen with ambiguous features […]” Sounds a bit like the old guy I buy my vodka from in the corner shop. I’d explain this a bit more (not my guy, who’s very nice, as it happens, but your specimens).

Line 498, “This ambiguous diatom may be a small valve ….” The specimen is not really ambiguous, it can’t be, the potential for interpretation is.

Line 605 et seq. the new species. I think this needs setting out differently. It needs a description first, then the discussion after. Perhaps this might work:

Cymbopleura subfrequens Reavie nov. spec. (Figs. 13S-X)
Valves of lanceolate, very slightly dorsiventral to symmetric along long axis, subtly triundulate with blunt, apiculate apices; length = 23-28 µm; width = 6-7 µm; striae = 14-18 in 10 µm. Axial area [sternum] narrow, linear-lanceolate, narrower at poles, widening gradually then merging with irregular to circular or oval central area. Central area comprises ⅓ to ½ of valve width. Raphe very weakly lateral, filiform at proximal ends, expanded but not visibly tipped toward either side. Distal raphe ends deflected dorsally. Striae weakly radiate at poles and central area, approaching parallel elsewhere.

Holotype “GLEI L. Huron, Hessel, MI, T1 SU coretop, McKay Creek EB, N45.992013, W084.393322, AR Kireta, EJ Tissier, 6/11/03”, Slide A. with etching A 6/11/03, slide deposited at NRRI, specimen represented by Fig. 13S.

[You will need a number to identify this slide].

The etymology of this newly described species acknowledges its similarity to C. frequens.

Similar specimens were observed from multiple locations around the Great Lakes, supporting the view that this is a new species. The more dorsiventral specimen (Fig. 13X) is presented as a possible variation of this species.

These specimens were originally identified as Cymbella incerta (Grunow) Cleve based on the broad depiction of that species by Krammer & Lange-Bertalot (1986). Further scrutiny of the valve shape and comparison with additional specimens for Cymbopleura incerta provided by
Krammer (2003) indicate that no published descriptions adequately characterize these Great Lakes specimens. Comparison can be made with Cymbopleura frequens Krammer and its varieties (Krammer, 2003), and Cymbopleura hybrida (Grunow ex Cleve) Krammer (Bahls, 2015), but those species have a coarser striae density and frequens has a more rostrate shape in the valve ends.

Line 634, “The genus name Delicata Krammer was replaced by Wynne (2019)”.
Do you need this? If so, a better explanation should be provided.

Line 636, Delicatophycus neocaledonicus (Krammer) Wynne (Figs. 11B-Q)
This was a replacement name so it is cited: Delicatophycus neocaledonicus Wynne

Line 704, “Krammer’s (1997b) description was determined to be a close fit to these specimens during GLEI assessments, but on further scrutiny they likely represent at least one undescribed species, or greatly increase the permissible striae density for E. evergladianum”. OK, well, how can one decide?

Line 868 and 879: Encyonopsis cf. krammeri .very fine. (Figs. 12LL-VV)
Encyonopsis cf. krammeri .very fine and narrow. (Figs. 12NNN-RRR)

Not sure I like this approach of adding qualifier to specimens. It seems like a lurch back to the days before Linnaeus when bionomials were unknown. But what to do? Well, just keep those observations in the descriptions.

Line 953, so is this salvation?
“Encyonopsis vandamii Krammer as presented by Krammer (1997b). As for most of these small Great Lakes Encyonopsis, this name should be considered tentative until genetic comparisons can be made against examples from the type locality, in this case Bavaria”. I’ve absolutely no idea why anyone would think this, as if there is some revealed wisdom that arises from genetic data, whatever that might be…

Experimental design

Comments in 1 above

Validity of the findings

Comments in 1 above

Additional comments

Comments in 1 above

Reviewer 2 ·

Basic reporting

As indicated in the review, there are several issues with the basic reporting. Some pertinent literature has not been cited, and the references section for the amphoroid and cymbelloid diatom treatments in particular seem to be dated. Please include more up-to-date references and discuss them. Figures 2A-LL are not readable. These results are not discussed at all. Many of the figures have litte to no information content. The figures need substantial revision.

Experimental design

No Comment.

Validity of the findings

Many of the identifications appear correct. But discussions about many of the taxa are lacking substantive, detailed elements.

Additional comments

The author has produced a “monograph” of what has been called the cymbelloid and gomphonemoid diatoms as well as the ‘amphoroid diatoms collected along the USA coastline of the 5 major Laurentian Great Lakes. The basis for this study was many (over 200) collections to examine the ecological conditions and associations of diatoms in those differing conditions. The plates for this publication were used to help develop a harmonious/consistent approach to the taxonomy of the diatoms encountered in this spatially (and ecologically) disparate set of sites. This extends the development of a much-needed diatom flora of the Great Lakes, produced by Reavie and colleagues over the last 7 years (starting the publication of Reavie & Kireta in 2015). A total of 154 taxa are treated in this work, including the description of 1 new taxon (not two, as indicated on line 130 of the manuscript). The approach seems to have two major focal points, first a systematic account of the taxa encountered in the study (as a ‘monograph’ as stated on line 11 of the manuscript) and the ecological information for common taxa (based on the description of the analysis of these data, provided starting on line 149).

While a treatment of this group of taxa from the Great Lakes is needed, the current manuscript will need a major revision before it can serve that role. I will list some general issues to start, then describe some of the finer issues that the author might consider.

First of all, the images used to document the taxa are quite poor. If this treatment is going to assist others in the identification of taxa from the Great Lakes (and the surrounding region), the images need to be better. These images look like they were taken with a poor microscope. The quality of the images, for even a single taxon are uneven (that is being generous). In fact, I would estimate that 5-10% of the images have almost no information content (maybe that they are of ‘diatoms’ may be all they offer). The percentage of poor-quality images goes up for some taxa (some of the smaller species, as might be expected), but even on plate 7 (of some big diatoms), three of those seven images (figs a, b, and e) would be difficult to use to identify a species. There are huge differences in contrast, image quality and specimen quality. The plates need to be modified to show clearly the taxa, their range of variability and features used for their identification. That is currently not the case. These plates have images that are not publication quality.

This is not a monograph. That work would involve a much deeper treatment of the taxa, offering data on the original description, homotypic synonyms as well as a view of past author’s perspective on heterotypic synonyms. That level of detail is not provided for most of the taxa here. For the amphoroid diatoms treated here, for example, the average number of cited references for each of the taxa is just 2. No types, exsiccatae specimens or other verified specimens were consulted. The places where there might be important observations, documentation or conclusions are not pursued. For example (and these are numerous in the manuscript), in discussing Amphora aequalis, the author states (lines 199-200), “This taxon agrees with Krammer’s (1980) depiction, although it was sometimes indistinguishable from A. inariensis in the same article.” In his treatment of A. inariensis the author states (lines 224-5), “This taxon agrees with Krammer’s (1980) depiction, although it was sometimes indistinguishable from A. aequalis in the same article.” The author then discusses whether the puncta are visible with LM in the two taxa. But, what was the trouble in distinguishing between the two species? Puncta visibility? If so, what type of lens is being used? Are some other features the problem? Were the two species examined with SEM? By the way, for the amphoroid diatoms there is an excellent treatment of them by Levkov et al. which is cited only once for this group.

Again, this superficial treatment of taxa flows across many taxa (here are just a few more examples, one per genus…):

*Cymbellla neocistula var. islandica (line 443); indicating that “…discerning the many taxa in the “Cymbella cistula” complex in the Great Lakes is challenging.” is not helpful unless you tell us why.

*Cymbopleura kuelbsii var. nonfasciata Krammer (line 556), “While Krammer (2003) present type specimens from France, this Great Lakes specimen is a fair fit to the species.” What does “fair fit” mean?

*Similar comments can be made for the first treatment of Encyonema silesiacum (line 781)

*What is the “overlap” claimed for the specimens identified as Encyonopsis cesatii (line 844) and E. cesatiformis?

For Gomphonema innocens (line 1122) we learn it is “…similar to G. parvulum…”. Says who? Whose concept of G. parvulum?

Regarding the new species description: The elements are included to conform to the Rules of Nomenclature for Algae, Fungi and Plants. The description could be formatted to make the separate items easier to find for readers, and called out as “Description” in the text. There are lots of examples in the literature as to how to do this. The beginning part might be better put into a ‘Diagnosis’ section. To follow best practice, the new species Cymbopleura subfrequens, should be documented with scanning electron microscopy. SEM images should be provided to document a new species that the author indicates has been found in several localities around the region (assuming that specimens are not so rare that they cannot be found in the SEM). I will note that for this species, 3 of the images (figures 13U, 13Wa, 13Wb) are not very informative and it seems hard to imagine that the specimen illustrated in 13V is the same taxon as those in 13S and 13T.

The author might note that the “Reimeria sinuata“ specimens illustrated with distinctly punctate [uniseriate] striae (Plate 16, figures I, J, P, Qa-c) are likely R. uniseriata Sala et al. 1993. A helpful guide to this genus is provided in Levkov & Ector 2010, Nova Hedwigia 90(3/4): 469-489 (“A comparative study of Reimeria species”).

The author might refer to a recently published paper on whether Encyonopsis evergladianum occurs in the Great Lakes region (Kociolek et al. 2021, Great Lakes Botanist 60: 24-55).

Regarding the ecological information provided for the common taxa in this group: The author has gone to great lengths to tell us the methods for treating ecological data, which has been delivered for each common taxon in Figures 2A-LL. Given all the information synthesized here (although condensed into a single plate, whose quality is such in the reviewer’s copy that the images become pixelated to a degree that the information is not readable), we might expect some detailed comments about the results. So, what are we provided with in the text to better understand the ecological conditions a particular taxon might be found in? For Amphora aequalis, for example, we get (lines 203-4), “Environmental characteristics for this species are provided in Fig. 2A.” That is it. I would say that if after all of the data analysis on ecological data that has happened, and there is only provided that statement and reference made only to a (in this case, unreadable) figure, either this is really not important (and this entire element ought to be dropped from the manuscript) or, if the author feels it is important, then there needs to be a fuller treatment of these data in the text of the manuscript.

There are many details for the author to take a look at (using consistently correct scientific notation, lots of spelling mistakes, details remaining from what must have been previous versions of this manuscript, etc.).

There is a need to address the important lack of an overall taxonomic treatment of the diatoms of the Laurentian Great Lakes. Especially for this group of diatoms. Unfortunately, in its present form, this manuscript is not that treatment. The manuscript needs a major revision and is not publishable in this form. I encourage the author to address these basic issues and offer an up-to-date identification guide that will support a wide range of users studying the Great Lakes and their surrounding watersheds.

Reviewer 3 ·

Basic reporting

1) Clear, unambiguous, professional English language used throughout?
No.
For example, lines 10-19, Abstract:
“This monograph contains descriptions of taxa from the diatom genera Amphora, Halamphora, Cymbella, Cymbopleura, Delicatophycus, Encyonema, Encyonopsis, Reimeria, Gomphonema, Gomphosphenia, Gomphonella, Gomphosinica, and Gomphoneis from periphytic and surface sediment samples in the coastal ecosystems of the Laurentian Great Lakes. Light micrographs are provided for diatom taxa recorded in 207 samples from 106 wetlands, embayments, high-energy and deep, nearshore locales of the five Great Lakes. 154 taxa are characterized, and 1 previously undescribed taxon is named. For 39 of the more common species, lake and habitat specificity, modeled optima for phosphorus and chloride and tolerance to coastal anthropogenic stressors are described. One new species is erected: Cymbopleura subfrequens Reavie nov. spec.”
Your monograph identified taxa from the …, not descriptions of taxa, because in most cases, you only provided the valve dimensions and stria density, but not gave more descriptions of morphology for each identified species.
One new species is described, not is erected, because this new species exists in the nature, you cannot erect it, but you can describe it.

For example, lines 201-203,
“Krammer gives length = 17-33 µm; width =202 3-6 µm; dorsal striae = 14-18/10 µm. Great Lakes specimens have length = 17-22 µm, width =203 3.5-4.5 µm, dorsal striae = 18-20/10 µm.”
The more rigorous and standardized statements may be “Krammer gives valve length range 17-33 µm; width range 3-6 µm; dorsal stria density 14-18/10 µm. Great Lakes specimens have valve length range 17-22 µm, width range 3.5-4.5 µm, dorsal stria density 18-20/10 µm.”. I.E., use valve length range, width range, and stria density (please see https://diatoms.org/, this American website uses valve length range, width range, and stria density).

2) Figures are relevant, high quality, well labelled & described?
No. The author provided only LM images and the figure quality not high. Since the diatom individual sizes vary largely for each species, a size diminution series of valves for each species is helpful and more valuable. Figures are not well labelled & described and do not follow the figure format of PeerJ [Please consult two references of PeerJ: Carlos Sánchez et al, 2019, Diatom identification including life cycle stages through morphological and texture descriptors; Cüneyt Nadir Solak et al, 2021, Nitzschia anatoliensis sp. nov., a cryptic diatom species from the highly alkaline Van Lake (Turkey)].

3) Raw data supplied?
I do not find the raw data.

Experimental design

1) Original primary research within Scope of the journal?
Yes.
2) Rigorous investigation performed to a high technical & ethical standard? Methods described with sufficient detail & information to replicate?
No. Please see also above “1. Basic reporting”. The author provided the valve dimensions and stria density but did not tell readers how many valves he has measured for each taxon. For high technical & ethical standard, the author needs to measure more than 30 different valves for each species so that the results reach the statistical significance and are more credible.

Validity of the findings

All underlying data have been provided; they are robust, statistical sound, & controlled?
No. Please see also above “2. Experimental design”. See below “new species checks” as well.

Additional comments

New species checks
Do you agree that it is a new species?
No.
Line 605: Author described a new species, Cymbopleura subfrequens Reavie nov. spec. (Figs. 13S-X). Author argued this new species remarkably similar to C. frequens, but there are other similar species with which the author did not compare. An example is Cymbopleura incertiformis, which has similar valve outline, valve length range 29.5-60.7 µm, width range 7.0-9.4 µm, Striae in 10 µm 14-17 at the valve center, 20-24 at the ends on the dorsal side (see https://diatoms.org/species/cymbopleura_incertiformis). The author exhibited seven valves of this new species (see Figure 13 S-X) and stated, “Because I observed similar specimens from multiple locations around the Great Lakes, a new species is erected.” (See line 620-621). Since you observed many specimens, why do not tell the readers how many specimens you have measured? As we know, the diatoms are small, single-celled organisms, and their valve lengths are largely variable, so Cymbopleura subfrequens may be a small population of C. inertiformis.

At this moment, many new diatom species are being described mostly without providing the DNA data for them, but mostly providing the SEM (scanning electric microscopy) images for them. In the genus Cymbopleura, the apical pore fields, the dorsally deflexed distal raphe fissures, and the shape of areolae, all need confirming by using the SEM images, so it is necessary to provide SEM images for Cymbopleura subfrequens in this manuscript, rather than wait for the future researchers to do the SEM examination. Furthermore, C. subfrequens may be a naviculoid diatom. All above questions can be addressed by using SEM images, so SEM investigations are needed.

---

## Round 0.2 · Major Revisions

Dear Dr. Reavie,

Despite reviewers finding your manuscript improved in several parts, in both round of revisions, some of them deem the SEM images of newly-described taxa as the norm in diatoms taxonomy, at least in peer-reviewed journals. For this reason, I ask you to add SEM images at least for the new taxa described in the manuscript.

Please, respond point-to-point to the comments of reviewers to speed up the process of revision. Once again, thank you for submitting your manuscript to PeerJ and we look forward to receiving your revision.

Sincerely,
Gabriele Casazza

·

Basic reporting

no comment

Experimental design

no comment

Validity of the findings

Only one thing at this stage:

the holotype specimen.

Holotype. Fig. 16R; slide deposited at NRRI: "GLEI L. Huron, Hessel, MI, T1 SU coretop, 247 McKay Creek EB, N45.992013, W084.393322, AR Kireta, EJ Tissier, 6/11/03 Slide A” with etching “A 6/11/03.”


I get this and it is probably acceptable but a figure is not the best way to do this. The figure must have been based on a specimen. What specimen? If I need to look at this again I cannot easily find the specimen in the illustration as it is not marked in any way, so the holotype immediately becomes a lectotype. So why not have the slide as the holotype and add in “The type is illustrated by Fig. 12R”. This way any specimens on the slide will be understood as 'part' of the holotype figure 12R being one.

Additional comments

no comment

Reviewer 2 ·

Basic reporting

See general comments

Experimental design

No Comment

Validity of the findings

See general comments

Additional comments

It is clear that the author read over all of the comments and in many cases made a good faith effort to review and then either revise or not the areas that received comments. There are still 3 areas of the manuscript that, in my opinion, deserve the attention of the author.

The LM’s are [still] not great. While I appreciate the methods the author and his team members employed to keep their taxonomy as consistent as possible, there is nothing to say that their in-house methods should be published. Despite addressing a few pictures, there are still many others that still do not even represent a good record of the species they are supposed to document.

The ecological figures: They have been broken into 4 figures instead of 1, which is an excellent start. But they still are not explained or discussed. If these figures were worth making and including in this paper, then they deserve more description and explanation. The authors reply that they are self-explanatory is not credible. These should be fully explained or deleted from the paper.

The lack of SEM images for the new species description was cited by two of the three reviewers as an issue. The author’s reply that Krammer did not do this for all the taxa he described is a red herring. Krammer’s book was not reviewed by peers. In this case, you are asking the peer community to review your work, and they are stating that the norm in diatoms is to include SEM images of newly-described taxa. Given the widespread nature of this taxon, that should be worth the effort.

If the author cannot address in a subsequent revision these three issues beyond what is presented here, then I can only recommend that the manuscript be rejected.

---

## Round 0.3 · Minor Revisions

Dear Dr. Reavie,

The reviewer feels that the use of light microscopy images alone reduces the quality of this work limiting its usefulness for many researchers in the future. However, they believe that your work advances the knowledge of a still little-known flora. Therefore, even if the main weakness cannot be addressed, they suggest some minor changes to improve the quality of the manuscript. Please, respond point-to-point to the comments of reviewers to speed up the process of revision. Once again, thank you for submitting your manuscript to PeerJ and we look forward to receiving your revision.
Sincerely,
Gabriele Casazza

Reviewer 2 ·

Basic reporting

Addressed previously; this review pertains to the most recent recent version of the manuscript.

Experimental design

Addressed previously; this review pertains to the most recent recent version of the manuscript.

Validity of the findings

Addressed previously; this review pertains to the most recent recent version of the manuscript.

Additional comments

The author has addressed 2 of the 3 issues raised in the previous review; The ecological data are described and presented in the taxon treatment, and (unfortunately) the new species proposal has been dropped since no SEM work can be undertaken by the author.

For the 3rd issue raised, related to the quality of the LM images, no further adjustments have been made. I am sorry that this is the case, as these images will detract significantly from the manuscript's use in the future. Some documentation is better than none, but this should not be the standard for this type of work.

I wonder if the author might tell us for the 154 taxa presented, how many (or what percentage of that number) could not be reliably identified.

And on line 125, the words "In most cases" should be dropped.

---

## Round 0.4 · accepted · Accept

Dear Dr. Reavie,

I am pleased to inform you that your paper "Asymmetric, biraphid diatoms from the Laurentian Great Lakes" is accepted for publication in the PeerJ. Congratulations!

Thank you for submitting your work to PeerJ.

Sincerely,
Gabriele Casazza