# Supplementary material for: Asymmetric, biraphid diatoms from the Laurentian Great Lakes
Source: PeerJ. 2023 Feb 14;11:e14887. doi: 10.7717/peerj.14887 (PMC9936871; doi:10.7717/peerj.14887)
Supplement: Supplemental Information 2 — For geomorphic types, HE, high-energy shoreline; CW, coastal wetland; EB, embayment; PW, protected wetland; RW, riverine wetland; nearshore, core surface samples collected from ~30 m depth near shore. Details of the methods used for water quality analyses are provided by Reavie et al. (2006). Missing values indicate they were not measured, except for Secchi depths where it indicates the location was too shallow to obtain a measurement. [file peerj-11-14887-s002.docx]

| Table S1 List of coastal sample locations and coordinates. For geomorphic types, HE = high-energy shoreline, CW = coastal wetland, EB = embayment, PW = protected wetland, RW = riverine wetland, nearshore = core surface samples collected from ~30 m depth near shore. Details of the methods used for water quality analyses are provided by Reavie *et al.* (2006). Missing values indicate they were not measured, except for Secchi depths where it indicates the location was too shallow to obtain a measurement. | | | | | | | | | | | | | | | | | | | |
| --- | --- | --- | --- | --- | --- | --- | --- | --- | --- | --- | --- | --- | --- | --- | --- | --- | --- | --- | --- |
| **Lake** | **Segment-shed** | **Latitude** | **Longitude** | **Sample type** | **Geomorphic type** | **pH** | **Temperature (°C)** | **Specific conductivity (µS/cm)** | **Turbidity (NTU)** | **Total suspended solids (mg/L)** | **Alkalinity (meq/L)** | **Chlorophyll *a* (µg/L)** | **Total phosphorus (µg/L)** | **Total nitrogen (µg/L)** | **Ammonium (µg/L)** | **Nitrates + nitrites (µg/L)** | **Dissolved organic carbon (mg/L)** | **Chloride (mg/L)** | **Secchi depth (m)** |
| Superior | 29 | 47.35 | -91.17 | epilithon | HE | 7.83 | 8.3 | 93.4 | 0.4 | 0.0 | 43.6 | 0.93 | 3 | 432 | 8 | 337 | 1.37 | 1.40 |  |
| Superior | 42 | 47.02 | -91.66 | epilithon | HE | 7.83 | 11.3 | 93.1 | 0.1 | 0.4 | 44.0 | 0.64 | 3 | 420 | 4 | 325 | 1.40 | 1.30 |  |
| Superior | 43 | 46.96 | -91.75 | core top | nearshore | 9.28 | 12.2 | 98.5 |  | 0.6 |  | 1.52 | 4 | 461 | 5 | 328 | 1.39 | 1.96 |  |
| Superior | 51 | 46.78 | -92.09 | epilithon | HE | 7.83 | 13.8 | 112.8 | 1.1 | 0.6 | 46.4 | 1.01 | 9 | 512 | 46 | 331 | 2.00 | 3.85 |  |
| Superior | 52 | 46.75 | -92.11 | core top | CW | 7.57 | 32.4 | 927.0 | 3.5 | 4.5 | 215.9 | 4.89 | 521 | 8060 | 6406 | 261 | 27.07 | 75.90 | 0.42 |
| Superior | 55 | 46.65 | -92.25 | core top | PW | 9.30 | 25.8 | 165.8 | 5.1 | 13.0 | 69.5 | 8.54 | 39 | 802 | 8 | 3 | 20.25 | 6.08 | 0.50 |
| Superior | 61 | 46.69 | -92.03 | core top | RW | 8.69 | 26.2 | 205.1 | 3.8 | 7.2 | 60.7 | 13.97 | 47 | 837 | 14 | 140 | 14.21 | 14.95 |  |
| Superior | 63 | 46.68 | -91.99 | core top | EB | 7.40 | 13.1 | 134.3 | 57.8 | 50.0 | 59.9 | 12.44 | 29 | 1035 | 21 | 367 | 14.45 | 14.04 | 0.19 |
| Superior | 68 | 46.68 | -91.82 | core top | RW | 7.39 | 19.6 | 130.4 | 19.9 | 10.0 | 49.1 | 1.50 | 82 | 1729 | 43 | 79 | 9.00 | 10.50 | 0.20 |
| Superior | 84 | 46.79 | -91.37 | core top | RW | 6.99 | 23.1 | 170.4 | 26.6 | 17.5 | 73.5 | 4.34 | 169 | 1541 | 45 | 148 | 10.00 | 7.50 | 0.22 |
| Superior | 88 | 46.86 | -91.13 | core top | PW | 7.64 | 22.4 | 191.3 | 4.2 | 3.6 | 89.0 | 1.87 | 14 | 237 | 4 | 2 | 6.21 | 0.90 |  |
| Superior | 102 | 46.59 | -90.94 | core top | CW | 7.80 | 19.3 | 126.7 | 2.9 | 6.3 | 54.2 | 2.09 | 21 | 293 | 11 | 143 | 1.87 | 1.80 | 2.20 |
| Superior | 104 | 46.58 | -90.94 | core top | RW | 7.54 | 17.2 | 170.4 | 8.0 | 11.4 | 82.8 | 1.12 | 42 | 158 | 7 | 28 | 2.87 | 3.36 | 0.64 |
| Superior | 105 | 46.58 | -90.91 | core top | PW | 7.71 | 19.4 | 126.0 | 7.2 | 10.2 | 57.7 | 2.33 | 37 | 311 | 5 | 125 | 2.17 | 2.61 | 0.79 |
| Superior | 109 | 46.66 | -90.72 | core top | CW | 7.83 | 24.4 | 92.5 | 7.1 | 8.3 | 42.2 | 3.84 | 17 | 283 | 3 | 0 | 5.67 | 1.59 | 0.62 |
| Superior | 110 | 46.66 | -90.69 | core top | PW | 7.56 | 24.1 | 79.6 | 1.7 | 3.1 | 33.6 | 7.34 | 29 | 687 | 7 | 0 | 16.58 | 2.72 | 1.18 |
| Superior | 180 | 47.46 | -88.06 | epilithon | HE | 7.90 | 8.9 | 92.2 | 2.0 | 0.0 | 45.0 | 0.53 | 4 | 393 | 1 | 339 | 1.37 | 1.38 |  |
| Superior | 183 | 47.37 | -87.94 | core top | HE | 7.97 | 20.3 | 93.2 | 2.7 | 2.3 | 44.0 | 0.67 | 6 | 412 | 5 | 323 | 1.62 | 1.45 |  |
| Superior | 191 | 46.82 | -88.47 | core top | HE | 7.32 | 20.8 | 96.8 | 0.7 | 2.0 | 42.1 | 0.67 | 7 | 424 | 13 | 292 | 1.83 | 2.00 |  |
| Superior | 192 | 46.76 | -88.48 | core top | PW | 7.86 | 21.5 | 173.4 | 1.9 | 2.4 | 90.9 | 0.40 | 14 | 111 | 1 | 6 | 2.09 | 0.78 |  |
| Superior | 200 | 46.9 | -88.15 | core top | PW | 7.47 | 23.5 | 70.9 | 2.0 | 4.0 | 35.7 | 5.87 | 10 | 416 | 9 | 29 | 8.76 | 1.15 |  |
| Superior | 202 | 46.89 | -87.87 | ponar | nearshore | 8.83 | 20.0 | 98.1 |  | 0.3 |  | 0.64 | 2 | 366 | 6 | 299 | 1.49 | 1.57 |  |
| Michigan | 204 | 45.04 | -87.05 | core top | PW | 7.17 | 21.7 | 107.4 | 3.3 | 2.5 | 48.3 | 2.30 | 10 | 288 | 11 | 47 | 6.57 | 1.05 |  |
| Michigan | 205 | 45.04 | -87.05 | core top | EB | 9.70 | 28.4 | 256.4 |  | 7.2 |  | 2.00 | 41 | 843 | 17 | 2 | 8.46 | 15.58 |  |
| Superior | 208 | 46.58 | -87.37 | core top | PW | 6.49 | 22.0 | 71.2 | 3.1 | 2.0 | 24.1 | 0.53 | 8 | 362 | 17 | 39 | 7.74 | 2.45 |  |
| Superior | 212 | 46.49 | -87.14 | core top | PW | 5.09 | 26.1 | 22.5 | 0.9 | 1.4 | 3.4 | 2.18 | 11 | 624 | 11 | 0 | 17.47 | 0.33 |  |
| Superior | 213 | 46.52 | -87.01 | epilithon | HE | 7.63 | 19.7 | 96.2 | 1.5 | 1.0 | 41.2 | 1.00 | 6 | 459 | 9 | 271 | 3.84 | 1.83 |  |
| Superior | 217 | 46.42 | -86.63 | core top | EB | 8.19 | 16.1 | 102.0 | 2.0 | 0.5 | 45.3 | 0.37 | 4 | 370 | 4 | 294 | 1.62 | 2.08 |  |
| Superior | 225 | 46.68 | -85.54 | ponar | nearshore | 8.70 | 18.8 | 98.4 |  | 0.7 |  | 0.51 | 2 | 429 | 9 | 305 | 1.46 | 1.56 |  |
| Huron | 242 | 46.17 | -84.21 | core top | CW | 7.87 | 22.6 | 216.1 | 26.5 | 32.0 | 103.7 | 4.23 | 99 | 1125 | 20 | 0 | 25.53 | 0.44 | 0.20 |
| Huron | 247 | 45.99 | -84.34 | core top | EB | 7.87 | 18.9 | 189.4 | 6.8 | 10.8 | 80.7 | 0.70 | 16 | 375 | 38 | 111 | 2.63 | 5.40 | 0.85 |
| Huron | 248 | 46 | -84.53 | core top | CW | 8.07 | 23.0 | 215.1 | 1.3 | 1.6 | 96.6 | 0.58 | 11 | 458 | 35 | 113 | 4.55 | 6.34 |  |
| Michigan | 269 | 45.94 | -86.25 | core top | CW | 7.95 | 17.4 | 223.5 | 2.8 | 5.0 | 86.2 | 2.89 | 16 | 502 | 17 | 153 | 7.23 | 6.70 |  |
| Michigan | 273 | 45.8 | -86.59 | core top | EB | 7.93 | 19.7 | 259.7 | 1.5 | 2.0 | 100.9 | 1.60 | 10 | 323 | 6 | 16 | 4.86 | 10.03 |  |
| Michigan | 275 | 45.81 | -86.78 | core top | CW | 8.53 | 24.5 | 252.2 | 2.8 | 7.1 | 100.4 | 0.35 | 16 | 317 | 12 | 38 | 5.89 | 10.01 | 1.50 |
| Michigan | 278 | 45.91 | -86.95 | core top | CW | 7.67 | 22.7 | 282.7 | 2.4 | 7.3 | 147.4 | 2.57 | 25 | 791 | 14 | 16 | 21.37 | 5.02 |  |
| Michigan | 281 | 45.7 | -87.08 | epilithon | RW | 8.44 | 23.7 | 263.3 | 0.5 | 2.5 | 134.1 | 1.25 | 15 | 760 | 11 | 30 | 19.77 | 2.54 |  |
| Michigan | 289 | 45.09 | -87.59 | core top | HE | 7.94 | 18.2 | 260.7 | 0.4 | 2.0 | 105.7 | 1.67 | 14 | 426 | 21 | 134 | 4.94 | 10.05 |  |
| Michigan | 290 | 45.04 | -87.61 | core top | CW | 7.23 | 20.8 | 197.4 | 3.3 | 2.2 | 82.7 | 1.47 | 74 | 2439 | 115 | 80 | 43.14 | 8.62 |  |
| Michigan | 294 | 44.89 | -87.83 | core top | CW | 8.29 | 21.3 | 284.8 | 9.8 | 31.7 | 111.3 | 28.39 | 69 | 959 | 40 | 39 | 4.92 | 13.94 | 0.57 |
| Michigan | 299 | 44.68 | -87.98 | core top | RW | 8.05 | 28.3 | 660.0 | 13.0 | 28.6 | 234.7 | 30.34 | 181 | 1465 | 25 | 365 | 10.65 | 40.90 | 0.37 |
| Michigan | 300 | 44.66 | -87.99 | core top | CW | 8.81 | 30.4 | 339.6 | 7.5 | 20.0 | 130.0 | 11.68 | 60 | 906 | 9 | 26 | 7.91 | 18.89 | 0.67 |
| Michigan | 302 | 44.62 | -88.01 | core top | PW | 8.63 | 23.1 | 347.8 | 80.8 | 260.8 | 124.7 | 85.66 | 397 | 2372 | 9 | 4 | 7.93 | 23.10 | 0.15 |
| Michigan | 304 | 44.56 | -88.03 | core top | CW | 8.28 | 26.8 | 365.8 | 14.1 | 45.0 | 136.8 | 39.10 | 159 | 1410 | 13 | 185 | 8.21 | 20.12 | 0.19 |
| Michigan | 305 | 44.53 | -87.98 | core top | RW | 8.94 | 26.8 | 443.4 | 32.4 | 53.0 | 154.5 | 75.00 | 314 | 1983 | 9 | 1 | 10.55 | 40.25 | 0.26 |
| Michigan | 311 | 44.84 | -87.54 | epilithon | EB | 8.82 | 24.6 | 265.8 | 2.4 | 6.4 | 100.4 | 4.54 | 21 | 441 | 4 | 4 | 4.53 | 12.45 |  |
| Michigan | 313 | 45.28 | -87.02 | epilithon | EB | 7.98 | 19.6 | 269.1 | 0.7 | 2.0 | 104.7 | 0.67 | 6 | 394 | 32 | 128 | 2.99 | 11.61 |  |
| Michigan | 325 | 44.14 | -87.57 | core top | RW | 8.17 | 25.3 | 661.0 | 35.2 | 90.0 | 247.9 | 90.45 | 405 | 2904 | 43 | 1005 | 14.24 | 17.52 | 0.17 |
| Michigan | 339 | 43.69 | -87.7 | core top | nearshore | 9.30 | 13.7 | 290.6 |  | 0.7 |  | 1.07 | 6 | 413 | 6 | 274 | 1.80 | 11.62 |  |
| Michigan | 350 | 42.62 | -87.81 | epilithon | HE | 8.36 | 22.2 | 297.9 | 1.6 | 11.5 | 109.4 | 1.07 | 15 | 458 | 10 | 249 | 2.05 | 11.04 |  |
| Michigan | 352 | 42.49 | -87.8 | core top | RW | 7.75 | 26.6 | 351.0 | 1.4 | 1.8 | 122.9 | 0.33 | 18 | 512 | 40 | 128 | 3.71 | 15.25 | 2.45 |
| Michigan | 354 | 42.24 | -87.78 | core top | nearshore | 9.05 | 12.7 | 292.1 |  | 1.0 |  | 1.02 | 2 | 411 | 13 | 313 | 1.71 | 11.81 |  |
| Michigan | 356 | 41.71 | -87.52 | core top | HE | 8.59 | 28.5 | 299.7 | 0.8 | 5.5 | 104.0 | 1.70 | 13 | 579 | 15 | 121 | 2.46 | 6.80 |  |
| Michigan | 361 | 41.8 | -86.73 | core top | HE | 8.29 | 22.8 | 295.5 | 6.2 | 10.5 | 109.4 | 4.94 | 6 | 430 | 12 | 261 | 1.97 | 12.67 |  |
| Michigan | 364 | 41.95 | -86.57 | core top | PW | 7.84 | 26.7 | 549.3 | 4.8 | 1.5 | 168.6 | 0.83 | 8 | 781 | 16 | 6 | 15.12 | 68.44 |  |
| Michigan | 365 | 42.09 | -86.48 | core top | RW | 8.09 | 22.4 | 517.0 | 3.8 | 11.0 | 168.7 | 5.47 | 46 | 1043 | 5 | 814 | 2.29 | 21.85 | 0.47 |
| Michigan | 369 | 42.39 | -86.28 | core top | RW | 8.18 | 24.3 | 458.9 | 4.9 | 7.1 | 117.0 | 6.67 | 57 | 1422 | 399 | 790 | 7.18 | 27.04 |  |
| Michigan | 374 | 42.88 | -86.21 | core top | RW | 8.23 | 20.3 | 325.3 | 1.7 | 4.3 | 112.1 | 1.62 | 25 | 817 | 18 | 535 | 3.07 | 17.99 |  |
| Michigan | 375 | 42.97 | -86.22 | core top | RW | 7.49 | 25.0 | 317.2 | 9.8 | 10.0 | 111.5 | 4.00 | 44 | 726 | 89 | 215 | 5.85 | 20.18 | 0.50 |
| Michigan | 378 | 43.17 | -86.3 | core top | RW | 8.33 | 24.2 | 475.8 | 7.4 | 28.0 | 133.5 | 27.50 | 227 | 2236 | 77 | 479 | 7.01 | 47.05 |  |
| Michigan | 388 | 43.98 | -86.47 | core top | HE | 8.35 | 14.7 | 296.4 | 1.1 | 3.0 | 119.8 | 2.54 | 11 | 654 | 9 | 407 | 2.63 | 12.48 |  |
| Michigan | 389 | 44.05 | -86.49 | ponar | nearshore | 8.95 | 18.7 | 293.2 |  | 1.3 |  | 1.45 | 2 | 363 | 10 | 265 | 1.81 | 11.76 |  |
| Michigan | 393 | 44.47 | -86.24 | core top | RW | 8.08 | 12.3 | 340.3 | 5.1 | 7.2 | 183.7 | 2.01 | 38 | 1002 | 18 | 596 | 6.90 | 3.63 |  |
| Michigan | 396 | 44.79 | -86.1 | epilithon | HE | 7.90 | 9.4 | 273.9 | 0.8 | 0.6 | 112.6 | 0.24 | 2 | 437 | 5 | 296 | 1.64 | 10.98 |  |
| Michigan | 407 | 45.64 | -85.03 | epilithon | HE | 8.45 | 14.2 | 246.8 | 0.7 | 0.6 | 98.6 | 0.63 | 4 | 390 | 9 | 217 | 2.01 | 9.50 |  |
| Huron | 411 | 45.65 | -84.42 | core top | PW | 7.91 | 13.9 | 225.4 | 2.8 | 4.0 | 109.4 | 2.54 | 17 | 677 | 8 | 1 | 18.18 | 8.69 |  |
| Huron | 412 | 43.53 | -83.19 | core top | CW |  |  |  |  |  |  | 5.70 | 27 | 793 | 21 | 20 | 7.59 |  |  |
| Huron | 416 | 45.43 | -83.82 | ponar | nearshore | 8.49 | 16.5 | 184.5 |  | 1.0 |  | 0.82 | 2 | 261 | 12 | 336 | 1.54 | 6.27 |  |
| Huron | 417 | 45.4 | -83.74 | core top | EB | 7.18 | 12.6 | 235.1 | 1.8 | 0.9 | 94.3 | 0.18 | 1 | 413 | 6 | 288 | 1.71 | 7.40 |  |
| Huron | 419 | 45.34 | -83.52 | epilithon | EB | 8.29 | 13.4 | 199.9 | 5.4 | 5.7 | 82.9 | 2.30 | 6 | 476 | 6 | 247 | 1.90 | 6.43 |  |
| Huron | 420 | 45.27 | -83.41 | core top | PW | 7.01 | 29.0 | 1332.0 | 3.1 | 3.0 | 109.1 | 0.87 | 6 | 386 | 18 | 71 | 2.46 | 76.02 |  |
| Huron | 421 | 45.1 | -83.31 | core top | CW | 8.07 | 20.7 | 1051.0 | 6.7 | 22.0 | 106.3 | 3.14 | 18 | 535 | 17 | 1 | 7.88 | 14.13 |  |
| Huron | 422 | 45.04 | -83.38 | core top | RW | 8.07 | 21.3 | 390.2 | 7.6 | 10.6 | 177.2 | 3.20 | 45 | 812 | 10 | 10 | 13.07 | 12.11 |  |
| Huron | 423 | 44.98 | -83.44 | core top | CW | 8.61 | 20.6 | 233.2 | 2.8 | 9.3 | 100.7 | 4.07 | 17 | 595 | 10 | 84 | 4.99 | 7.67 |  |
| Huron | 425 | 44.77 | -83.29 | core top | HE | 8.20 | 24.6 | 200.4 | 1.7 | 1.2 | 74.2 | 1.30 | 5 | 393 | 18 | 244 | 1.51 | 6.29 |  |
| Huron | 426 | 44.52 | -83.31 | core top | HE | 8.28 | 17.8 | 214.8 | 3.5 | 1.6 | 89.1 | 1.51 | 11 | 443 | 14 | 261 | 2.08 | 6.89 |  |
| Huron | 439 | 43.99 | -83.81 | core top | CW | 8.32 | 16.2 | 366.7 | 8.0 | 12.0 | 180.3 | 4.54 | 36 | 779 | 11 | 332 | 7.42 | 14.05 |  |
| Huron | 445 | 43.89 | -83.91 | core top | CW | 8.60 | 23.9 | 333.4 | 11.0 | 11.8 | 86.1 | 4.48 | 25 | 692 | 26 | 47 | 7.38 | 32.30 |  |
| Huron | 446 | 43.87 | -83.91 | core top | CW | 8.30 | 22.5 | 328.0 | 19.3 | 9.4 | 97.7 | 5.91 | 27 | 642 | 24 | 13 | 6.23 | 34.60 |  |
| Huron | 451 | 43.72 | -83.94 | core top | PW | 8.60 | 26.8 | 634.0 | 8.6 | 10.0 | 113.7 | 13.95 | 47 | 1744 | 20 | 13 | 20.38 | 99.10 |  |
| Huron | 453 | 43.63 | -83.8 | top core | RW | 8.08 | 26.2 | 180.0 | 4.3 | 16.0 | 173.3 | 12.95 | 64 | 1291 | 96 | 863 | 7.39 | 112.63 |  |
| Huron | 459 | 43.87 | -83.34 | core top | CW | 8.77 | 19.8 | 336.0 | 4.8 | 5.0 | 76.6 | 3.74 | 23 | 707 | 20 | 14 | 7.00 | 37.60 |  |
| Huron | 461 | 43.91 | -83.4 | core top | CW | 8.89 | 22.7 | 306.6 | 10.1 | 14.0 | 72.5 | 6.94 | 23 | 724 | 12 | 101 | 5.14 | 37.50 |  |
| Huron | 462 | 43.94 | -83.27 | core top | HE | 8.61 | 26.5 | 239.5 | 8.4 | 5.9 | 83.1 | 2.47 | 9 | 356 | 5 | 146 | 2.32 | 10.78 |  |
| Huron | 470 | 44.02 | -82.79 | epilithon | HE | 8.80 | 27.4 | 230.4 | 3.7 | 1.3 | 81.9 | 0.42 | 9 | 460 | 24 | 183 | 2.57 | 10.46 |  |
| Huron | 486 | 43.75 | -82.61 | epilithon | CW | 8.32 | 17.2 | 217.5 | 4.9 | 1.1 | 83.1 | 0.22 | 3 | 437 | 18 | 279 | 1.92 | 7.92 |  |
| Erie | 568 | 42.04 | -83.18 | core top | RW | 8.16 | 23.8 | 1131.0 | 2.5 | 11.0 | 168.1 | 11.08 | 50 | 494 | 8 | 0 | 6.28 | 98.94 |  |
| Erie | 569 | 42.01 | -83.2 | core top | RW | 7.49 | 21.3 | 1888.0 | 7.7 | 82.4 | 119.3 | 20.83 | 113 | 859 | 10 | 0 | 5.90 | 68.35 | 0.15 |
| Erie | 574 | 41.89 | -83.33 | core top | nearshore | 8.70 | 25.1 | 289.7 |  | 4.9 |  | 7.40 | 25 | 638 | 31 | 348 | 2.95 | 13.69 |  |
| Erie | 581 | 41.75 | -83.45 | core top | EB | 8.83 | 25.4 | 349.3 | 12.3 | 32.8 | 96.9 | 12.56 | 64 | 832 | 10 | 136 | 4.60 | 24.93 | 0.43 |
| Erie | 582 | 41.74 | -83.47 | core top | PW | 8.07 | 23.9 | 496.0 | 28.0 | 82.0 | 122.7 | 46.73 | 241 | 1330 | 32 | 14 | 7.42 | 45.10 | 0.15 |
| Erie | 585 | 41.69 | -83.44 | core top | EB | 8.78 | 23.6 | 343.6 | 56.7 | 69.6 | 111.4 | 73.96 | 84 | 2291 | 5 | 1429 | 6.96 | 20.59 | 0.10 |
| Erie | 588 | 41.62 | -83.19 | core top | PW | 8.40 | 23.1 | 326.0 | 51.6 | 90.0 | 101.8 | 25.90 | 77 | 3416 | 14 | 2610 | 5.68 | 18.38 | 0.10 |
| Erie | 591 | 41.54 | -83 | core top | HE | 8.27 | 25.4 | 281.1 | 9.8 | 3.0 | 78.9 | 2.00 | 17 | 819 | 44 | 248 | 3.65 | 18.60 |  |
| Erie | 593 | 41.5 | -82.8 | core top | PW | 7.52 | 28.9 | 581.0 | 1.3 | 6.5 | 203.8 | 12.37 | 117 | 2311 | 18 | 11 | 29.05 | 39.80 |  |
| Erie | 595 | 41.44 | -83.01 | core top | RW | 8.38 | 25.3 | 651.0 | 5.8 | 22.7 | 114.3 | 40.23 | 148 | 6210 | 32 | 6380 | 7.23 | 37.18 | 0.25 |
| Erie | 596 | 41.43 | -83 | core top | RW | 8.37 | 25.1 | 651.0 | 5.7 | 26.8 | 114.3 | 52.73 | 127 | 7265 | 67 | 6015 | 7.42 | 37.26 | 0.25 |
| Erie | 602 | 41.46 | -82.79 | core top | PW | 8.78 | 29.1 | 566.0 | 4.5 | 10.2 | 3.0 | 8.68 | 51 | 1411 | 3 | 8 | 16.06 | 43.46 | 0.20 |
| Erie | 605 | 41.43 | -82.65 | core top | EB | 9.02 | 30.0 | 340.3 | 12.2 | 42.0 | 89.7 | 25.63 | 124 | 980 | 12 | 14 | 6.07 | 25.10 | 0.30 |
| Erie | 606 | 41.42 | -82.62 | top core | CW | 8.84 | 25.8 | 470.0 | 15.1 | 18.6 | 106.9 | 11.88 | 51 | 592 | 19 | 28 | 6.37 | 50.68 | 0.20 |
| Erie | 610 | 41.38 | -82.51 | core top | RW | 7.58 | 24.9 | 477.7 | 15.1 | 27.0 | 152.0 | 16.42 | 51 | 1921 | 139 | 1146 | 7.35 | 39.13 | 0.30 |
| Erie | 617 | 41.5 | -81.7 | core top | HE | 8.12 | 16.9 | 320.5 | 18.3 | 24.2 | 100.1 | 1.47 | 36 | 1237 | 46 | 922 | 2.85 | 36.75 | 0.25 |
| Erie | 620 | 41.38 | -82.51 | core top | EB |  |  |  |  |  |  |  |  |  |  |  |  |  |  |
| Erie | 622 | 41.75 | -81.28 | core top | CW | 8.44 | 24.9 | 288.9 | 5.3 | 4.0 | 85.5 | 6.61 | 26 | 758 | 5 | 479 | 3.48 | 21.52 |  |
| Erie | 635 | 41.9 | -80.79 | core top | RW | 7.34 | 18.7 | 345.3 | 6.8 | 6.5 | 125.0 | 3.49 | 42 | 945 | 172 | 588 | 5.08 | 28.35 | 0.41 |
| Erie | 645 | 42.12 | -80.15 | core top | nearshore | 8.47 | 17.2 | 272.8 |  | 1.3 |  | 1.54 | 7 | 741 | 16 | 403 | 1.97 | 18.00 |  |
| Erie | 646 | 42.14 | -80.07 | core top | EB | 8.85 | 26.5 | 295.8 | 2.0 | 5.5 | 84.3 | 14.15 | 39 | 761 | 30 | 25 | 4.43 | 23.93 |  |
| Erie | 658 | 42.57 | -79.13 | core top | nearshore | 8.49 | 15.9 | 283.2 |  | 1.6 |  | 1.51 | 4 | 552 | 15 | 290 | 2.02 | 20.17 |  |
| Ontario | 688 | 43.37 | -78.17 | ponar | nearshore | 8.52 | 10.3 | 298.5 |  | 0.7 |  | 3.10 | 2 | 312 | 12 | 310 | 1.95 | 23.13 |  |
| Ontario | 696 | 43.3 | -77.71 | core top | CW | 8.62 | 27.2 | 427.5 | 9.1 | 10.2 | 127.6 | 18.29 | 79 | 729 | 13 | 2 | 6.07 | 106.57 | 0.40 |
| Ontario | 697 | 43.29 | -77.68 | core top | PW | 8.23 | 27.3 | 652.0 | 23.0 | 23.2 | 120.5 | 39.52 | 148 | 2393 | 352 | 63 | 8.50 | 106.57 | 0.25 |
| Ontario | 698 | 43.28 | -77.65 | core top | RW | 7.89 | 27.1 | 768.0 | 7.7 | 8.6 | 153.0 | 5.07 | 36 | 673 | 5 | 3 | 8.06 | 120.74 |  |
| Ontario | 700 | 43.26 | -77.62 | core top | RW | 7.63 | 27.8 | 420.0 | 3.6 | 1.3 | 88.7 | 1.20 | 91 | 1093 | 171 | 508 | 4.09 | 47.00 | 0.41 |
| Ontario | 719 | 43.29 | -76.89 | ponar | nearshore | 8.46 | 8.1 | 311.8 |  | 0.6 |  | 2.44 | 5 | 745 | 2 | 408 | 1.87 | 25.24 |  |
| Ontario | 720 | 43.3 | -76.84 | core top | PW | 6.70 | 23.2 | 263.4 | 1.4 | 2.8 | 114.3 | 1.96 | 51 | 1056 | 15 | 5 | 17.22 | 11.76 | 0.75 |
| Ontario | 723 | 43.35 | -76.68 | core top | PW | 8.05 | 25.2 | 195.2 | 1.1 | 2.7 | 87.6 | 2.53 | 19 | 682 | 16 | 7 | 9.16 | 4.03 |  |
| Ontario | 735 | 43.52 | -76.24 | core top | RW | 6.48 | 24.2 | 247.0 | 0.0 | 2.8 | 58.3 | 2.54 | 104 | 725 | 22 | 21 | 10.35 | 22.37 | 0.69 |
| Ontario | 739 | 43.61 | -76.19 | top core | HE | 8.39 | 22.8 | 257.9 | 7.4 | 16.9 | 59.8 | 8.28 | 35 | 533 | 12 | 234 | 3.24 | 27.55 |  |
| Ontario | 740 | 43.64 | -76.18 | core top | EB | 8.11 | 24.3 | 191.0 | 1.0 | 1.3 | 51.9 | 1.07 | 19 | 356 | 40 | 30 | 8.44 | 14.20 |  |
| Ontario | 743 | 43.67 | -76.18 | epilithon | CW | 8.73 | 24.1 | 206.1 | 4.0 | 3.6 | 63.0 | 2.27 | 27 | 511 | 8 | 2 | 4.09 | 20.84 |  |
| Ontario | 744 | 43.71 | -76.2 | ponar | nearshore | 8.47 | 11.0 | 331.8 |  | 1.1 |  | 3.21 | 3 | 624 | 2 | 414 | 2.26 | 28.04 |  |
| Ontario | 746 | 43.9 | -76.15 | epilithon | EB | 8.48 | 19.2 | 270.7 | 1.7 | 0.9 | 78.5 | 0.20 | 5 | 408 | 18 | 291 | 2.84 | 26.23 |  |
| Ontario | 747 | 43.97 | -76.06 | core top | CW | 8.88 | 24.7 | 209.8 | 3.7 | 7.7 | 73.6 | 9.88 | 48 | 637 | 26 | 14 | 7.51 | 10.66 | 0.47 |
| Ontario | 748 | 43.99 | -76.06 | core top | EB | 8.39 | 21.3 | 110.8 | 3.4 | 3.9 | 39.0 | 1.78 | 30 | 738 | 19 | 133 | 6.42 | 8.55 |  |
| Ontario | 755 | 44.06 | -76.15 | core top | EB | 9.16 | 25.0 | 254.1 | 10.2 | 8.3 | 75.1 | 4.27 | 44 | 479 | 25 | 10 | 3.73 | 11.70 | 0.61 |
| Ontario | 760 | 44.05 | -76.31 | core top | RW | 9.02 | 24.2 | 274.7 | 4.8 | 11.3 | 85.2 | 4.54 | 35 | 445 | 12 | 71 | 3.44 | 18.59 | 0.74 |
| Ontario | 761 | 44.07 | -76.33 | epilithon | EB | 8.73 | 21.4 | 287.5 | 3.1 | 1.3 | 83.2 | 1.91 | 8 | 464 | 11 | 213 | 2.95 | 19.55 |  |
